# Supplementary material for: HIVprotI: an integrated web based platform for prediction and design of HIV proteins inhibitors
Source: J Cheminform. 2018 Mar 9;10:12. doi: 10.1186/s13321-018-0266-y (PMC5845081; doi:10.1186/s13321-018-0266-y)
Supplement: Supplementary file 2 — Additional file 2. Source code of HIVProtI web server. [file 13321_2018_266_MOESM2_ESM.zip › HIVprotI_Source-code/hivprotI/weka-3-6-6/doc/weka/gui/beans/BeanCommon.html]

BeanCommon


---


|  |  |  |
| --- | --- | --- |
| **Overview** \| **Package** \| **Class** \| **Tree** \| **Deprecated** \| **Index** \| **Help** \| **Weka's home** | |  |
| **PREV CLASS**   **NEXT CLASS** | **FRAMES**    **NO FRAMES**     **All Classes** |  |
| SUMMARY: NESTED \| FIELD \| CONSTR \| METHOD | DETAIL: FIELD \| CONSTR \| METHOD |  |


---


## weka.gui.beans Interface BeanCommon

**All Known Implementing Classes:**: AbstractDataSink, AbstractEvaluator, AbstractTestSetProducer, AbstractTrainAndTestSetProducer, AbstractTrainingSetProducer, Associator, ClassAssigner, Classifier, ClassifierPerformanceEvaluator, ClassValuePicker, Clusterer, ClustererPerformanceEvaluator, CostBenefitAnalysis, CrossValidationFoldMaker, Filter, IncrementalClassifierEvaluator, InstanceStreamToBatchMaker, Loader, MetaBean, PredictionAppender, Saver, SerializedModelSaver, StripChart, TestSetMaker, TextViewer, TrainingSetMaker, TrainTestSplitMaker

---

``` public interface BeanCommon ```

Interface specifying routines that all weka beans should implement
in order to allow the bean environment to exercise some control over the
bean and also to allow the bean to exercise some control over connections.
Beans may want to impose constraints in terms of
the number of connections they will allow via a particular
listener interface. Some beans may only want to be registered
as a listener for a particular event type with only one source, or
perhaps a limited number of sources.

**Since:**
:   1.0

**Version:**
:   $Revision: 4806 $

**Author:**
:   Mark Hall

---

| **Method Summary** | |
| --- | --- |
| `boolean` | `connectionAllowed(java.beans.EventSetDescriptor esd)`             Returns true if, at this time, the object will accept a connection via the supplied EventSetDescriptor |
| `boolean` | `connectionAllowed(java.lang.String eventName)`             Returns true if, at this time, the object will accept a connection via the named event |
| `void` | `connectionNotification(java.lang.String eventName, java.lang.Object source)`             Notify this object that it has been registered as a listener with a source for recieving events described by the named event This object is responsible for recording this fact. |
| `void` | `disconnectionNotification(java.lang.String eventName, java.lang.Object source)`             Notify this object that it has been deregistered as a listener with a source for named event. |
| `java.lang.String` | `getCustomName()`             Get the custom (descriptive) name for this bean (if one has been set) |
| `boolean` | `isBusy()`             Returns true if. |
| `void` | `setCustomName(java.lang.String name)`             Set a custom (descriptive) name for this bean |
| `void` | `setLog(Logger logger)`             Set a logger |
| `void` | `stop()`             Stop any processing that the bean might be doing. |

**Method Detail**

### setCustomName

```
void setCustomName(java.lang.String name)
```

:   Set a custom (descriptive) name for this bean

    :   **Parameters:**: `name` - the name to use

---


### getCustomName

```
java.lang.String getCustomName()
```

:   Get the custom (descriptive) name for this bean (if one has been set)

    :   **Returns:**: the custom name (or the default name)

---


### stop

```
void stop()
```

:   Stop any processing that the bean might be doing.

---


### isBusy

```
boolean isBusy()
```

:   Returns true if. at this time, the bean is busy with some
    (i.e. perhaps a worker thread is performing some calculation).

    :   **Returns:**: true if the bean is busy.

---


### setLog

```
void setLog(Logger logger)
```

:   Set a logger

    :   **Parameters:**: `logger` - a `weka.gui.Logger` value

---


### connectionAllowed

```
boolean connectionAllowed(java.beans.EventSetDescriptor esd)
```

:   Returns true if, at this time,
    the object will accept a connection via the supplied
    EventSetDescriptor

    :   **Parameters:**: `esd` - the EventSetDescriptor **Returns:**: true if the object will accept a connection

---


### connectionAllowed

```
boolean connectionAllowed(java.lang.String eventName)
```

:   Returns true if, at this time,
    the object will accept a connection via the named event

    :   **Parameters:**: `eventName` - the name of the event **Returns:**: true if the object will accept a connection

---


### connectionNotification

```
void connectionNotification(java.lang.String eventName,
                            java.lang.Object source)
```

:   Notify this object that it has been registered as a listener with
    a source for recieving events described by the named event
    This object is responsible for recording this fact.

    :   **Parameters:**: `eventName` - the event: `source` - the source with which this object has been registered as a listener

---


### disconnectionNotification

```
void disconnectionNotification(java.lang.String eventName,
                               java.lang.Object source)
```

:   Notify this object that it has been deregistered as a listener with
    a source for named event. This object is responsible
    for recording this fact.

    :   **Parameters:**: `eventName` - the event: `source` - the source with which this object has been registered as a listener


---


|  |  |  |
| --- | --- | --- |
| **Overview** \| **Package** \| **Class** \| **Tree** \| **Deprecated** \| **Index** \| **Help** \| **Weka's home** | |  |
| **PREV CLASS**   **NEXT CLASS** | **FRAMES**    **NO FRAMES**     **All Classes** |  |
| SUMMARY: NESTED \| FIELD \| CONSTR \| METHOD | DETAIL: FIELD \| CONSTR \| METHOD |  |


---
